# Identification of Bacterial Pathogens in Organic Food of Animal Origin in Poland

**DOI:** 10.3390/foods13213526

**Published:** 2024-11-04

**Authors:** Maciej Sosnowski, Kinga Wieczorek, Jacek Osek

**Affiliations:** Department of Food Safety, National Veterinary Research Institute, Partyzantow 57, 24-100 Pulawy, Poland; maciej.sosnowski@piwet.pulawy.pl (M.S.); kinga.wieczorek@piwet.pulawy.pl (K.W.)

**Keywords:** organic food, food safety, foodborne pathogens, *Salmonella*, *Listeria monocytogenes*, *Campylobacter*, *Staphylococcus aureus*, STEC

## Abstract

The consumption of organic food has increased in recent years. In organic rearing animals are exposed to outdoor conditions, which may increase their risk of infection from various pathogens. In the present study the occurrence of the most significant foodborne pathogenic bacteria in organic meat and ready-to-eat organic meat products was assessed. Out of 100 raw organic meat samples tested, 72 were contaminated with bacterial pathogens. The highest percentage of contaminated samples was observed in poultry meat (92.5%) followed by pork meat (66.7%). Furthermore, 50.0% of beef origin samples were positive for the bacteria tested. *L. monocytogenes* was found in 39.0% of samples, *S. aureus* was identified in 37.0%, *Campylobacter* in 20.0%, *Salmonella* in 8.0% and Shigatoxin-producing *E. coli* in 4.0% of raw meat samples. In 31.0% of samples a co-occurrence of two (83.9%) or three (16.1%) pathogens was observed. Among 100 samples of organic meat products tested, only *L. monocytogenes* was found in 5.0% of samples. The result of the present study indicated that organic food may be a source of harmful microorganisms that may pose foodborne infections to consumers.

## 1. Introduction

Nowadays, organic food seems to be an essential element of healthy and sustainable lifestyles. The consumption of organic meat, dairy products, fruits and vegetables has increased worldwide in recent years, especially in developed countries [1,2]. Consumers are willing to pay higher prices for products which are of better quality and safer for health. Organic foods often contain higher concentrations of beneficial compounds, such as polyphenols and antioxidants, which may have anti-cancer properties and better nutritional attributes [3,4]. Moreover, organic products are increasingly chosen by consumers to avoid risk related to the consumption of food with artificial additives, pesticides, herbicides, growth hormones and similar adverse substances. It also has a positive influence on agro-biodiversity (breeds used by the farmers) and natural biodiversity (wild life) [5,6].

In the European Union (EU), the production of organic food is regulated by legal acts, with the most important being Regulation No. 2018/848 [7]. EU organic food producers must also ensure compliance with the relevant microbiological criteria described in Commission Regulation No 2073/2005 on microbiological criteria for foodstuffs [8]. In the United States, organic production is regulated by the U.S. Department of Agriculture (USDA) [9]. A key distinction between conventional and organic production systems is that the organic system offers animals outdoor access. During organic rearing, livestock has access to fresh air and agricultural lands. Although such a system is more friendly for animal welfare, it may pose a higher risk of exposure to zoonotic pathogens from the environment [10,11]. The risk of contamination at the farm level may increase due to the use of organic fertilizers and irrigation water containing fecal bacteria [12,13]. It should be added that outdoor system production together with a limited use of artificial fertilizers and pesticides as well as restricting the application of antibiotics may not provide a strict control of microbial pathogens spread by food-producing animals [14]. It has been shown that some organic foods have been linked to foodborne outbreaks [15,16]. Differences in the prevalence of foodborne bacterial pathogens in conventional and alternative meat production systems have been reported; however, the findings have been inconsistent [17,18,19].

It is often difficult to establish the relationship between data and a type of farm production because of many confounders. Some authors reported that the incidence of pathogenic bacteria in organic animals as well as in organic food products may be higher than in conventional ones. Conversely, Lucke [12] concluded from analyses of the data of other investigations that organic foods do not seem to be at greater risk than conventional ones when good agricultural practice rules are implemented on the farm [20].

Foodborne pathogens such as *Salmonella* and *Campylobacter* cause thousands of human infections every year in the EU. According to a recent European Food Safety Authority (EFSA) zoonoses report, in 2022 137,107 campylobacteriosis and 65,208 salmonellosis infections were reported in humans. *Listeria monocytogenes*, with 2738 cases reported in 2022, induces severe disease with high rates of fatality and hospitalization. Food poisoning caused by enterotoxins produced by *Staphylococcus aureus* or Shiga toxins produced by *Escherichia coli* (STEC) also affect many people (2199 and 7117 cases reported in 2022, respectively) [21]. However, the above data refer to infections caused by the consumption of food of animal origin in general, without specifying organic food. Meat and products of meat origin are an important source of foodborne infections and one of the most significant links between food-producing animals and humans [14,21]. Taking the above into account, a potential presence of bacterial pathogens has been indicated as a special challenge for organic food production schemes [22]. Although most of the epidemiological data in the literature and information included in Rapid Alert System for Food and Feed (RASFF) refer to specific types of meat, details of the farming practices from which the meat was obtained are rarely specified. Since 2020, there have been seven reports of bacterial pathogens (*Salmonella* or Shigatoxin-producing *E. coli*) in organic meat in the RASFF system. None of the notifications was from Poland. The microbiological safety of organic food depends on the presence of microorganisms in the environment or on the secondary pathogen contamination of the products, which may occur at further stages of the food chain. However, the sources of microbiological contamination are usually present at primary production [23].

The aim of the present study was to assess the occurrence of the most significant foodborne pathogenic bacteria, i.e., *Salmonella*, *L. monocytogenes*, *S. aureus*, *Campylobacter* and Shigatoxin-producing *E. coli* (STEC) in organic meat and ready-to-eat (RTE) products originating from organic meat.

## 2. Materials and Methods

### 2.1. Food Samples

A total of 200 samples, including 100 samples of raw meat and 100 samples of RTE meat products, were examined. Among the raw meat samples, 40 were of poultry origin, and the remaining 60 samples were pork and beef meat (30 of each).

RTE meat products included 46 samples of smoked meats (gammon, bacon, pork neck, pork shoulder, sirloin, ham, pastrami), 45 samples of steamed and smoked sausages, six samples of steamed white sausages, two samples of raw sausages and one sample of offal sausage (Polish name salceson).

According to the type of meat, 73 RTE samples were from pork, 24 samples were of poultry origin, and the remaining three samples were beef. The samples were collected from seven voivodships (administrative districts) across Poland, obtained from producers certified in compliance with Regulation 2018/848 [7]. The products were labelled with the organic production logo of the European Union, complying with the model presented in the Regulation. They had Polish certification authority numbers: PL-EKO-01, PL-EKO-04, PL-EKO-07 and PL-EKO-09. The purchased samples of 500 ± 100 g weight, vacuum packed, were transported to the laboratory at 1–8 °C over a maximum of 24 h. After reception at the laboratory, the samples were stored at 1–5 °C up to 24 h before testing.

### 2.2. Isolation and Identification of Pathogenic Bacteria

#### 2.2.1. *Listeria monocytogenes*

*L. monocytogenes* was detected according to the ISO 11290-1 Standard [24]. Briefly, 25 ± 0.1 g of the sample was homogenized in 225 mL of half Fraser broth (Bio-Rad, Hercules, CA, USA) and incubated at 30 ± 1 °C for 24–26 h. One hundred µL of the culture was transferred into 10 mL of Fraser broth (Bio-Rad, Hercules, CA, USA) and incubated for 24 ± 2 h at 37 ± 1 °C. *L. monocytogenes* was isolated on Ottaviani-Agosti and Palcam agar plates (Bio-Rad, Hercules, CA, USA), respectively. Presumptive *L. monocytogenes* colonies were subcultured on TSYEA agar (Oxoid, Basingstoke, Hampshire, UK) at 37 ± 1 °C for 24 ± 2 h. Sugar fermentation (xylose and rhamnose), catalase, hemolysis and CAMP tests were performed as described [24]. The isolates that biochemically corresponded to *L. monocytogenes* were subjected to further molecular identification with multiplex PCR to determine *L. monocytogenes* molecular serogroups as described previously [25,26].

#### 2.2.2. *Campylobacter*

*Campylobacter* was identified according to the ISO 10272-1 Standard [27]. A sample portion of 10 ± 0.1 g was added to 90 mL of Bolton broth (Oxoid, Basingstoke, Hampshire, UK), homogenized and incubated at 41.5 ± 1 °C for 44 ± 4 h under microaerophilic conditions using Campygen (Thermo Fisher Scientific, Waltham, MA, USA). The cultures were then streaked onto mCCD agar (Oxoid, Basingstoke, Hampshire, UK) and modified Karmali agar (Oxoid, Basingstoke, Hampshire, UK) containing a selective supplement (amphotericin B, cefoperazone, sodium pyruvate and vancomycin) and incubated at 41.5 ± 1 °C under microaerophilic conditions for 44 ± 4 h. The putative *Campylobacter* isolates were selected based on their growth characteristics and colony morphology. The oxidase activity test, bacterial morphology, motility and aerobic growth at 25 ± 1 °C were performed. The bacterial isolates that biochemically corresponded to *Campylobacter* were subjected to PCR species identification as described previously [28].

#### 2.2.3. *Salmonella*

*Salmonella* was detected according to ISO 6579-1 Standard [29]. In short, a 25 ± 0.1 g sample was pre-enriched in 225 mL of Buffered Peptone Water (Oxoid, Basingstoke, Hampshire, UK) for 18 ± 2 h at 34–38 °C. After incubation, 0.1 mL of the culture was transferred into 10 mL of Rappaport Vassiliadis soya (Bio-Rad, Hercules, CA, USA), and 1 mL into 10 mL of Mueller-Kauffmann (Bio-Rad, Hercules, CA, USA) broths, and each culture was then incubated for 24 ± 3 h at 41.5 °C and 34–38 °C, respectively. The cultures were then streaked onto Xylose Lysine Deoxycholate (Oxoid, Basingstoke, Hampshire, UK) and Rapid’Salmonella agar plates (Bio-Rad, Hercules, CA, USA), respectively, and incubated for 24 ± 3 h at 34–38 °C. Presumptive *Salmonella* colonies were streaked on nutrient agar and further incubated at 37 ± 1 °C for 24 ± 3 h. Bacterial biochemical confirmation was performed using Vitek 2 (bioMérieux, Lyon, France). *Salmonella* was serotyped according to the White–Kauffmann–Le Minor scheme by slide agglutination with specific O- and H-antigen sera (Sifin Diagnostics, Berlin, Germany).

#### 2.2.4. *Staphylococcus aureus*

*S. aureus* was detected according to the ISO 6888-3 Standard [30]. A 10 ± 0.1 g sample portion was enriched in 90 mL of double concentrated Giolitti-Cantoni broth (Oxoid, Basingstoke, Hampshire, UK) for 24–48 h at 37 ± 1 °C. The cultures were then streaked onto Baird-Parker agar with rabbit plasma fibrinogen (Bio-Rad, Hercules, CA, USA) and incubated at 37 ± 1 °C for 24–48 h. Typical colonies of *S. aureus*, round, smooth, convex, moist, grey to black, often with a pale edge, surrounded by an opaque zone and often with an outer transparent zone, were picked up, streaked on nutrient agar and incubated at 37 ± 1 °C for 24 ± 3 h.

#### 2.2.5. Shigatoxin-Producing *Escherichia coli*

Shigatoxin-producing *E. coli* (STEC) was detected according to the ISO 13136 Standard [31]. Briefly, a 25 ± 0.1 g sample was pre-enriched in 225 mL of Buffered Peptone Water (Oxoid, Basingstoke, Hampshire, UK) for 18–24 h at 37 ± 1 °C. After incubation, multiplex real-time PCR targeting virulence genes eae, stx1 and stx2 was conducted in a 20 μL reaction volume using the following reaction mixture: 1 X TaqMan^®^ Universal PCR Master mix (Thermo Fisher, Waltham, MA, USA), 400 nM each of forward and reverse primers, 100 nM of each labeled probes and 2 μL DNA template. The real-time-PCR thermal cycling was conducted using a CFX96 system (Bio-Rad, Hercules, CA, USA) with the following cycling parameters: 95 °C for 10 min for the initial denaturation of DNA and activation of the hot-start Taq polymerase, followed by 40 cycles of amplification at 95 °C for 15 s and 60 °C for 60 s. Samples testing positive for the presence of the stx1 and/or stx2 genes were tested for *E. coli* O103, O111, O145, O157, O26 and O104 serogroup-associated genes. When the stx1 and/or stx2 genes were detected, the isolation of the strain from the enrichment sample broth was attempted. Enriched samples were plated on Tryptone Bile X-Glucuronide (TBX, Oxoid, Basingstoke, Hampshire, UK) agar and incubated for 18–24 h at 37 ± 1 °C. Up to 50 colonies with *E. coli* morphology were picked up and point-inoculated on nutrient agar. Pools of 10 colonies were tested by real-time PCR for the presence of virulence genes eae, stx1 and stx2; afterward, colonies from positive pools were tested singularly in order to identify STEC strains. Additionally, the O-type and H-type genes of isolated STEC were determined using the methodology described by Wieczorek and Osek [32].

## 3. Results

### 3.1. Contamination of Raw Meat with Bacterial Pathogens

Bacterial culture analyses revealed that 72.0% of raw organic meat samples were contaminated with at least one bacterial pathogen tested. The highest percentage of positive samples was observed in poultry meat, where among 40 samples pathogens were found in 37 (92.5%). For pork, bacteria were identified in 20 out of 30 samples tested (66.7%). The lowest percentage of samples containing pathogens was found for beef, where 15 out of 30 samples (50.0%) were positive. The remaining 28.0% of meat samples were free of the microorganisms tested. Such meat samples were of beef (15 out of 28, 53.6%), pork (10, 35.7%) and poultry origin, respectively (3, 10.7%).

**Figure 1:**
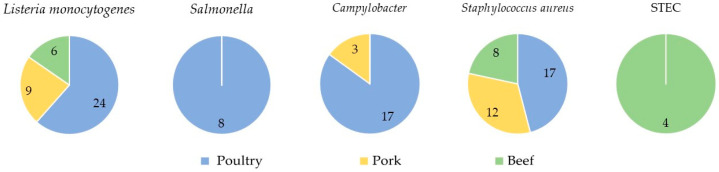
Distribution of bacterial pathogens in relation to the organic meat type tested. The numbers on the graphs indicate the number of strains isolated from particular types of meat.

**Figure 2:**
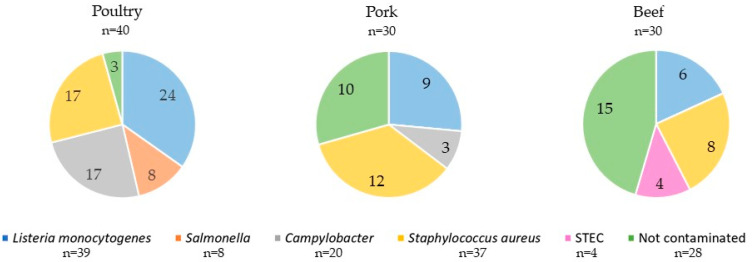
Presence of bacterial pathogens identified in particular types of organic meat. The numbers on the graphs indicate the number of contaminated samples of a particular type of meat with pathogens tested or number of samples free from pathogens.

The distribution of the detected microorganisms in relation to the type of organic meat is presented in Figure 1. The number of samples contaminated with particular microorganisms or free from the microorganisms for each type of meat is shown in Figure 2.

#### 3.1.1. Prevalence and Molecular Serotyping of *L. monocytogenes*

*L. monocytogenes* was found in 39.0% of all tested samples of raw meat. The most contaminated was poultry meat: 24 out of 39 isolates (61.5%) were recovered from this type of meat. The remaining isolates were obtained from pork (9; 23.1%) and beef (6, 15.4%).

All 39 *L. monocytogenes* isolates from raw meat were further tested towards molecular serogroups. Most isolates (20; 51.3%) were classified into the IIa serogroup; they were mainly from poultry meat (18 out of 20 isolates; 90.0%). The remaining two *L. monocytogenes* isolates of IIa were identified in beef meat. Seventeen of the 39 isolates (43.6%) were classified into the IIc serogroup. Eight (47.1%) of them were isolated from pork meat, five (29.4%) from poultry meat and four (23.5%) from beef. Serogroups IIb and IVb were represented by single isolates (2.6% each of them) and were from raw pork and poultry meat, respectively.

#### 3.1.2. Detection of *S. aureus*

The second most frequently detected bacterial pathogen was *S. aureus*, which was identified in 37 (37.0%) of raw meat samples. This microorganism was found in all types of meat tested. Most of the strains were isolated from poultry (17 out of 37, 46.0%), followed by pork (12, 32.4%) and beef (8, 21.6%).

#### 3.1.3. Identification of *Campylobacter*

*Campylobacter* was detected in 20 (20.0%) samples of raw meat. Most of them were identified in poultry meat (17 out of 20, 85.0%), and the remaining three samples were from pork (15.0%). None of the beef meat samples was contaminated with these bacteria. PCR of species identification showed that *C. coli* was found more frequently than *C. jejuni*—in 12 (60.0%), and in 8 (40.0%) samples, respectively. Both *C. coli* and *C. jeuni* were isolated from poultry meat (11 and six strains, respectively) and from pork (one and two strains, respectively).

#### 3.1.4. Prevalence and Serotyping of *Salmonella*

*Salmonella* was detected in eight raw meat samples (8.0%), all of which were poultry (20.0%). Serological tests showed that three isolates belonged to serovar *Salmonella* Enteritidis and three to serovar *S.* Indiana, whereas the remaining two isolates were *S.* Infantis.

#### 3.1.5. Detection and Molecular Serotyping of STEC

Shigatoxin-producing *E. coli* was detected in four samples of raw meat (4.0%). In another sample (1.0%) the presence of this pathogen was presumptive, i.e., only the stx1 and/or stx2 genes were identified, but the bacteria were not isolated. All five STEC-positive and presumptive positive samples were of beef origin. Three STEC isolates possessed the stx1 gene, while the remaining one strain had the stx2 gene. It was also shown that none of the bacteria tested was classified into the O157, O111, O26, O103 or O145 serogroup.

#### 3.1.6. Co-Occurrence of the Pathogens in Raw Meat Samples

**Figure 3:**
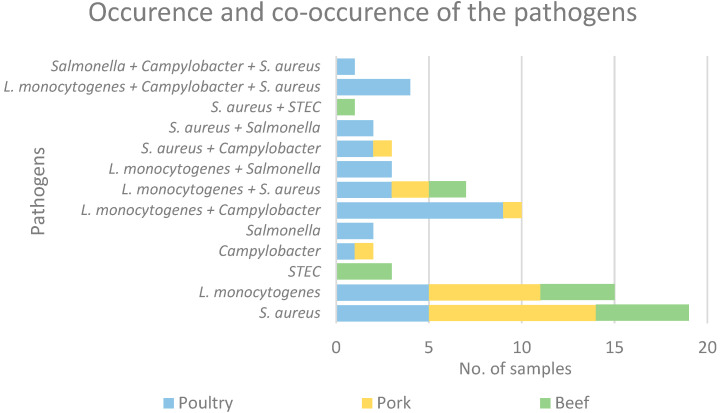
Occurrence of bacterial pathogens in organic raw meat tested.

In 31 (31.0%) samples of raw meat tested, the co-occurrence of two (26, 83.9% samples) or three (five, 16.1% samples) pathogens was observed (Figure 3). The most frequently occurring were identified as *L. monocytogenes* and *Campylobacter* (14 of 31, 45.2%), followed by *L*. *monocytogenes* and *S. aureus* (11, 35.5%), *Campylobacter* and *S. aureus* (eight, 25.8%) and *L*. *monocytogenes* and *Salmonella* (three, 9.7%). Furthermore, the simultaneous prevalence of three pathogens was identified in five samples: *L. monocytogenes*, *Campylobacter* and *S. aureus* (four samples, 12.9%) as well as *Salmonella*, *Campylobacter* and *S. aureus* (one sample, 3.2%). All five samples contaminated with the three pathogens were of poultry meat origin. Meat containing two pathogens simultaneously was also most frequently of poultry origin (19 out of 26, 73.1%), followed by beef (four, 15.4%) and pork (3three, 11.5%). On the other hand, 41 (41.0%) samples were contaminated with only one pathogen: 16 (39.0%) samples of pork, 13 (31.7%) of poultry and 12 (29.3%) of beef.

### 3.2. Presence of Bacterial Pathogens in RTE Meat Products

Among the 100 samples of organic meat products tested, only *L. monocytogenes* was found in five (5.0%) samples. This pathogen was present in three samples of sausages, including one raw (white) and two smoked sausages. The bacteria were also detected in one offal product and in one smoked meat. The RTE products contaminated with *L. monocytogenes* were of pork meat (four samples) and poultry meat (one sample) origin, respectively.

All *L. monocytogenes* isolates recovered from RTE meat products were further tested for molecular serogroups. It was found that four out of five isolates were classified into the IIa serogroup. Among them, three isolates were recovered from pork sausages and another one from poultry sausage. One strain of the IIc serogroup was isolated from pork smoked shoulder.

## 4. Discussion

In the present study, the occurrence of five significant foodborne bacterial pathogens, *Salmonella*, *Listeria monocytogenes*, *Staphylococcus aureus*, *Campylobacter* and Shigatoxin-producing *E. coli* (STEC), was examined. *L. monocytogenes* and *S. aureus* were detected most frequently in organic meat, whereas in organic RTE food only *L. monocytogenes* was observed. The other bacteria tested were found less frequently and only in raw meat.

Many studies have examined the difference in pathogen prevalence between organic and conventional meat production systems, but the results are often contradictory [19,33]. It should be underlined that many times reliable comparison is impossible because of the various organic production requirements established in different countries as well as the type of samples and methods used for the pathogens’ detection. However, in terms of *Campylobacter* most researchers agree that this microorganism is more common in organically farmed poultry than conventional poultry. Engvall [34] and Heuer et al. [33] showed the presence of *Campylobacter* in 100% of organic broiler flocks compared to 36.7% and 10.0% in conventional poultry batches, respectively. A comparison of the occurrence of *Campylobacter* in organic and conventional broiler carcasses carried out in Denmark revealed a significantly higher prevalence of these bacteria in organic meat [18]. However, the results obtained by Economou et al. [17] have not indicated any differences among free-range and conventional poultry samples. In the current study, 20.0% of raw meat samples and 42.5% of poultry meat examined were *Campylobacter*-positive. According to data from the recent EU zoonoses report, this pathogen was noted in 11.6% of fresh meat, and meat from broilers and turkeys showed the highest percentages of contamination—12.0% and 11.2%, respectively [21]. Many factors influence higher prevalences of *Campylobacter* in organic conditions, but the most important seems to be the outdoor husbandry system, which is much less controllable than the conventional one. The age of broilers slaughtered in organic production, which is usually over 81 days, may also be a factor, as it is higher than in conventional farming and the risk of infection has been shown to increase with the age of broilers [33]. On the other hand, Voidarou et al. [19] have not found *Campylobacter* in either conventionally or free-grazing broiler carcasses. At the same time, they detected *Salmonella* and *L. monocytogenes* in organic meat, while the poultry meat from conventional production was free from these pathogens. Because the organic samples were taken directly from rural households the authors suggest that the low microbiological quality of this kind of meat was due to insufficient hygiene in the slaughter conditions [19].

In the current investigations, isolated *L. monocytogenes* was further characterized towards molecular serogroups. The vast majority of isolates was classified to serogroups IIa and IIc, which were also characteristic for *L. monocytogenes* of food origin in other studies, including those performed in Poland [35,36,37,38]. It should be underlined that isolates representing the IIa serogroup are often responsible for listeriosis in human [39,40].

The average level of *Salmonella*-positive samples in the EU in 2022 was established on 2.1% of samples in the non-ready-to-eat food category. The percentages of contaminated meat and meat products from broilers and turkeys were 5.1% and 3.3%, respectively [21]. In the present investigation, the proportion of samples with *Salmonella* was significantly higher than those reported by EU countries, i.e., 20%. However, the number of samples tested was rather low and not comparable with that investigated in the EU. A similar trend was observed by Mazengia et al. [41] regarding raw poultry meat collected in retail markets in the USA. Likewise, a higher prevalence of STEC was observed in the current study compared to the overall EU data. This pathogen was detected in 13.3% (4 out of 30 tested) beef meat samples in the present study, whereas in the EU 4.3% of meat from bovine sources were found to be contaminated with STEC [21]. However, it is not stated whether the EU samples originated from organic food.

According to regulation No. 2073/2005, *L. monocytogenes* should be tested in various RTE foods, in particular foods that may favor the growth of bacteria [8]. In the EU, the rate of positive RTE meat and meat products samples was up to 2.6% compared to 5.0% in organic foods tested in the current investigation [21]. Additionally, this study showed a high percentage of organic raw meat samples contaminated with *L. monocytogenes* (39.0%). Miranda et al. [14] also observed high prevalence of this bacteria in organic meat, at 49.1%, and this was a higher level than in non-organically sourced meat (41.0%).

Furthermore, the level of *S. aureus* in the present study was also found to be high, i.e., 37.0%, and in case of poultry meat it was even higher—42.5%. A higher level of *S. aureus* contamination was observed in studies conducted by Miranda et al. [14], who identified these bacteria in 37 out of 55 (67.3%) samples of organic reared poultry meat and in 35 out of 61 (57.4%) samples of conventional ones. The other data, which refer to the occurrence of coagulase-positive *Staphylococcus* in fresh cheese, have not shown a dissimilarity between the production systems, and *S. aureus* was detected in all organic and conventional samples tested [42].

There are not many investigations towards comparisons of the microbiological contamination of organic and conventional foods in Poland. There are no Polish data regarding the occurrence of foodborne pathogens in organic raw meat and meat products either. However, the study conducted by Berthold et al. [43] showed that the microbiological quality of various types of cheese from ecological production was lower than conventional products. The opposite results were obtained by Kukułowicz [44], who concluded that there were no differences in the average number of microorganisms in food in relation to the type of production. Although the current study concerns the situation in Poland and might not give a global overview of the safety of organic food, it highlights a problem connected with this type of food.

Prache et al. [4] remarked that the safety of organic meat depends on the accumulation of environmental contaminants or chemical residues during animals’ life. Due to keeping animals for a longer time on farms with free access to outdoors, they are exposed to environmental contaminants, which results in a higher bioaccumulation in animals and finally in the meat. Likewise, the risk of infection by bacterial pathogens is enhanced. Additionally, fertilization practices, including natural manure applied in organic farms, may also increase the risk of contamination in organic products [13]. On the other hand, conventional production systems involving cages in the case of poultry and enclosed buildings with slatted floors in the case of pigs and cattle, as well as the use of industrial growth feeds, effectively increase productivity. However, stress on animals reared under such conditions may reduce meat quality and properties. Stress factors are related to rearing conditions such as stocking density and the temperature and ventilation system, as well as the health status of the animals, the duration of rearing, transport conditions and distance to the slaughterhouse. At high animal concentration, high levels of heat and ammonia are produced, negatively affecting the welfare of the animals kept [45,46].

For organic food safety and reducing the risk of microbial contamination, it is fundamental to increase the food operators’ knowledge on principles of Hazard Analysis and Critical Control Points (HACCP), together with the implementation of good hygiene practices (GHP) in relation to the special requirements of organic production [47]. A study performed by Nazareth et al. [16] on the prevalence of *Salmonella* and *E. coli* O157: H7 in organically raised cattle showed that respecting food safety strategies can significantly reduce the contamination of animals and meat with bacterial pathogens.

Organic food is more expensive and is considered healthier and safer for consumers. However, this study has shown that the consumption of these foods can be potentially dangerous due to a relatively high contamination rate with foodborne pathogens, which requires the strict control of the production chain. Similar conclusions were drawn by Harvey et al. [48], who reported some outbreaks due to *S.* Enteritidis, *E. coli* O157:H7 or *Campylobacter* associated with organic raw meat, cream or eggs in the US.

## 5. Conclusions

This study concludes that organic meat and meat products can serve as a source of potentially pathogenic microorganisms. It was shown that several samples were contaminated with *L. monocytogenes* and *S. aureus*. The consumption of such products contaminated with harmful bacteria may pose a public health risk by causing serious foodborne infections. Furthermore, the results of our study, as well as those from other authors, indicate that organic production cannot prevent food contamination by pathogenic microorganisms and that organic food products are characterized by a similar or even higher level of contamination with bacterial pathogens as conventional products.

## Data Availability

The original contributions presented in the study are included in the article, further inquiries can be directed to the corresponding author.
